# Identification of Specific Cell Subpopulations and Marker Genes in Ovarian Cancer Using Single-Cell RNA Sequencing

**DOI:** 10.1155/2021/1005793

**Published:** 2021-10-07

**Authors:** Yan Li, Juan Wang, Fang Wang, Chengzhen Gao, Yuanyuan Cao, Jianhua Wang

**Affiliations:** ^1^Department of Obstetrics and Gynecology, The Yancheng Clinical College of Xuzhou Medical University, The First People's Hospital of Yancheng, Yancheng, 224001 Jiangsu, China; ^2^Department of Obstetrics and Gynecology, The First Affiliated Hospital of Soochow University, Suzhou, 215006 Jiangsu, China; ^3^Department of Gastroenterology, The Yancheng Clinical College of Xuzhou Medical University, The First People's Hospital of Yancheng, Yancheng, 224001 Jiangsu, China

## Abstract

**Objective:**

Ovarian cancer is the deadliest gynaecological cancer globally. In our study, we aimed to analyze specific cell subpopulations and marker genes among ovarian cancer cells by single-cell RNA sequencing (RNA-seq).

**Methods:**

Single-cell RNA-seq data of 66 high-grade serous ovarian cancer cells were employed from the Gene Expression Omnibus (GEO). Using the Seurat package, we performed quality control to remove cells with low quality. After normalization, we detected highly variable genes across the single cells. Then, principal component analysis (PCA) and cell clustering were performed. The marker genes in different cell clusters were detected. A total of 568 ovarian cancer samples and 8 normal ovarian samples were obtained from The Cancer Genome Atlas (TCGA) database. Differentially expressed genes were identified according to ∣log2fold change (FC) | >1 and adjusted *p* value <0.05. To explore potential biological processes and pathways, functional enrichment analyses were performed. Furthermore, survival analyses of differentially expressed marker genes were performed.

**Results:**

After normalization, 6000 highly variable genes were identified across the single cells. The cells were divided into 3 cell populations, including G1, G2M, and S cell cycles. A total of 1,124 differentially expressed genes were identified in ovarian cancer samples. These differentially expressed genes were enriched in several pathways associated with cancer, such as metabolic pathways, pathways in cancer, and PI3K-Akt signaling pathway. Furthermore, marker genes, STAT1, ANP32E, GPRC5A, and EGFL6, were highly expressed in ovarian cancer, while PMP22, FBXO21, and CYB5R3 were lowly expressed in ovarian cancer. These marker genes were positively associated with prognosis of ovarian cancer.

**Conclusion:**

Our findings revealed specific cell subpopulations and marker genes in ovarian cancer using single-cell RNA-seq, which provided a novel insight into the heterogeneity of ovarian cancer.

## 1. Introduction

Ovarian cancer is one of the most common gynaecological cancers in the world, with high heterogeneity and poor prognosis [1]. High-grade serous ovarian cancer is the deadliest subtype of ovarian cancer, with up to 80% of patients recurring after initial treatment [2]. Despite advances in treatments such as surgery and chemotherapy, the 5-year survival rate of patients with advanced ovarian cancer remains around 30%-40% [3, 4]. Since ovarian cancer patients are usually diagnosed at an advanced stage, genetic risk prediction and prevention strategies will be an important way to reduce ovarian cancer mortality [5]. Targeted therapies significantly improve the therapeutic effects of patients with ovarian cancer [6]. However, ovarian cancer shows heterogeneity within the tumor that may affect the therapeutic outcomes of targeted therapies. Tumors including ovarian cancer usually consist of heterogeneous cells that are different in many biological features, like morphology, apoptosis, and invasion [7]. However, RNA-seq data reflect the average expression levels of different cells, not to reveal the intrinsic expression differences between different cell subpopulations. The genetic heterogeneity of ovarian cancer has been confirmed at single-cell resolution. The heterogeneity of gene expression levels greatly affects the patients' clinical outcomes [8]. Therefore, understanding the heterogeneity of tumors at the transcriptome level and the precise characterization of gene expression in tumors may help to identify better therapeutic molecular targets [9]. The characterization of heterogeneous tumor features will help to develop more effective molecular targeted therapeutics.

The basic unit of cancer is the innovative single cell along with genetics and epigenetics. Single-cell control determines the parameters of various aspects of cancer biology. Thus, single-cell analysis provides the ultimate resolution for us to understand the biology of various diseases [10]. Single-cell RNA-seq has been become a promising approach for revealing the clonal genotype and population structure of human cancers. RNA-seq of the single cell can be used to analyze the cell type in the tumor microenvironment, the tumor heterogeneity, and its clinical significance [11]. Unlike traditional sequencing methods, single-cell sequencing methods provide different types of omics analysis for individual cells, such as genomics and transcriptomics [12]. Among them, single-cell RNA sequencing (scRNA-seq) is capable of measuring gene expression at the single-cell level. Based on classical markers, the scRNA-seq reveals the heterogeneity of gene expression in individual cells or cells with the same type [13], rather than simply examining differential expression between two cells. In this study, we analyzed the heterogeneity among ovarian cancer cells and identified marker genes by scRNA-seq.

## 2. Materials and Methods

### 2.1. Ovarian Cancer Single-Cell RNA-seq Datasets

Single-cell RNA-seq gene expression data of ovarian cancer were employed from the Gene Expression Omnibus (GEO; https://www.ncbi.nlm.nih.gov/geo/) database with accession number GSE123476. According to the study of Winterhoff et al., 19 cells were excluded due to poor cell morphology, extremely large or small cell size, or evidence of multiple cells in the well. Meanwhile, 7 cells that did not express at least 1,000 of the highly expressed genes were also removed [14]. As a result, 26 ovarian cancer cells with low quality were removed from 92 cells. The barcode information and single-cell RNA-seq gene expression matrix were extracted for further analyses [14].

### 2.2. Quality Control Filtering and Data Normalization

The gene expression matrix was imported into the Seurat package in R (version 3.1.0; http://satijalab.org/seurat/). Seurat, as a tool for single-cell genomics, is used for quality control, analysis, and exploration of single-cell RNA-seq data [15]. For single-cell RNA-seq data, there could be cells with low quality, probably due to the loss of cytoplasmic RNA when the cells were disrupted. Since mitochondria were much larger than single transcriptional molecules, they were not easily leaked out of the broken cell membrane, causing the abnormally high proportion of mitochondrial genes among the cells in sequencing results. Thus, to remove cells with low quality, quality control was performed. After quality control, fragments per kilobase of transcript per million mapped read (FPKM) values were transformed into the log-space.

### 2.3. Detection of Highly Variable Genes across the Single Cells

To eliminate the dimensional relationship between variable genes and make the data comparable, using the NormalizeData function of the Seurat package, data were normalized with the log-normalize method. For each gene, we calculated the standard variance in all cells using the FindVariableFeature function. Herein, mean-variance was calculated as 1. Standard variance cut-off of 1 was used to identify highly variable genes. The top 20 highly variable genes were identified.

### 2.4. Cell Clustering Analysis Using Seurat

Principal component analysis (PCA) is a multivariate statistical method that examines the correlation between different variables. PCA was used to examine how to reveal the internal structure between multiple variables through a few principal components. That is, a few principal components were derived from the original variables while they retained the information of the original variables as much as possible and were not related to each other. In our study, PCA was carried out based on highly variable genes. Using the screened PCs as input, the cell clustering was visualized using Uniform Manifold Approximation and Projection (UMAP) via the RunUMAP function.

### 2.5. Gene Scoring

The CellCycleScoring function of the Seurat package was used to score the marker genes in the two cell cycles G2M and S based on the gene expression levels. We calculated the average expression value of S phase genes and G2/M phase genes for each cell. All genes were divided into different bins based on the average expression levels, and then, the control genes were randomly selected as the background from each bin. The average expression levels of these control genes were calculated. The average expression levels of control genes were subtracted from the average expression levels of S phase genes and G2/M phase genes to obtain S.Score and G2M.Score. S.Score < 0 and G2M.Score < 0 were judged as G1 phage, otherwise, which phage was judged as which score was higher. The difference between the cell cluster and the cell cycle distribution was examined by Fisher's test. The top ten differentially expressed genes and the cell cycle were separately plotted, which were visualized into heatmaps.

### 2.6. Detection of Marker Genes and Functional Enrichment Analysis

The cluster marker genes with ∣log2fold change (FC) | ≥0.25, the expression ratio of cell population ≤ 0.25, and *p* value ≤0.05 were identified using the “FindAllMarkers” function in the Seurat package. An expression heatmap was generated for given cells and genes using the DoHeatmap. The expression level of markers in each cluster was calculated, and the putative identities of each cell clustering were identified. The top 20 markers were plotted for each cluster. To explore potential biological processes and pathways enriched by markers in each cluster, functional enrichment analyses were performed using the gProfiler package.

### 2.7. Reconstruction of Differentiation Trajectories Using Monocle Analysis

The pseudotime estimation analysis of epithelial cancer cells and stromal cells was performed using the Monocle package. A pseudotime plot was generated that can account for both branched and linear differentiation processes based on the top 2000 highly variable marker genes.

### 2.8. Differential Expression Analysis and Function Enrichment Analysis Using Ovarian Cancer Datasets

A total of 593 ovarian cancer samples were obtained from The Cancer Genome Atlas (TCGA) using the UCSC Xena (https://tcga.xenahubs.net), including gene expression profiles and clinical information. Supplementary table 1 listed the IDs of all samples. After removing 17 relapse ovarian cancer, 568 ovarian cancer samples and 8 normal ovarian tissue samples were employed for this study. Differential expression analysis was then performed according to ∣log2FC | >1 and adjusted *p* value <0.05 using the limma package in R. To explore potential biological processes and pathways, functional enrichment analyses of upregulated and downregulated genes were performed using the gProfiler package in R, including Gene Ontology (GO) and Kyoto Encyclopedia of Genes and Genomes (KEGG). The GO terms include biological process (BP), cellular component (CC), and molecular function (MF). Terms with *p* value <0.05 were significantly enriched.

### 2.9. Overall Survival Analysis

Marker genes and differentially expressed genes were overlapped. Overall survival and recurrence-free survival analyses of differentially expressed marker genes were performed. Kaplan-Meier survival curves and log-rank tests were performed to evaluate the associations between ovarian cancer prognosis and the expression of these prognostic genes.

## 3. Results

### 3.1. Identification of Three Cell Subpopulations across Ovarian Cells Based on Single-Cell RNA-seq

**Figure 1:**
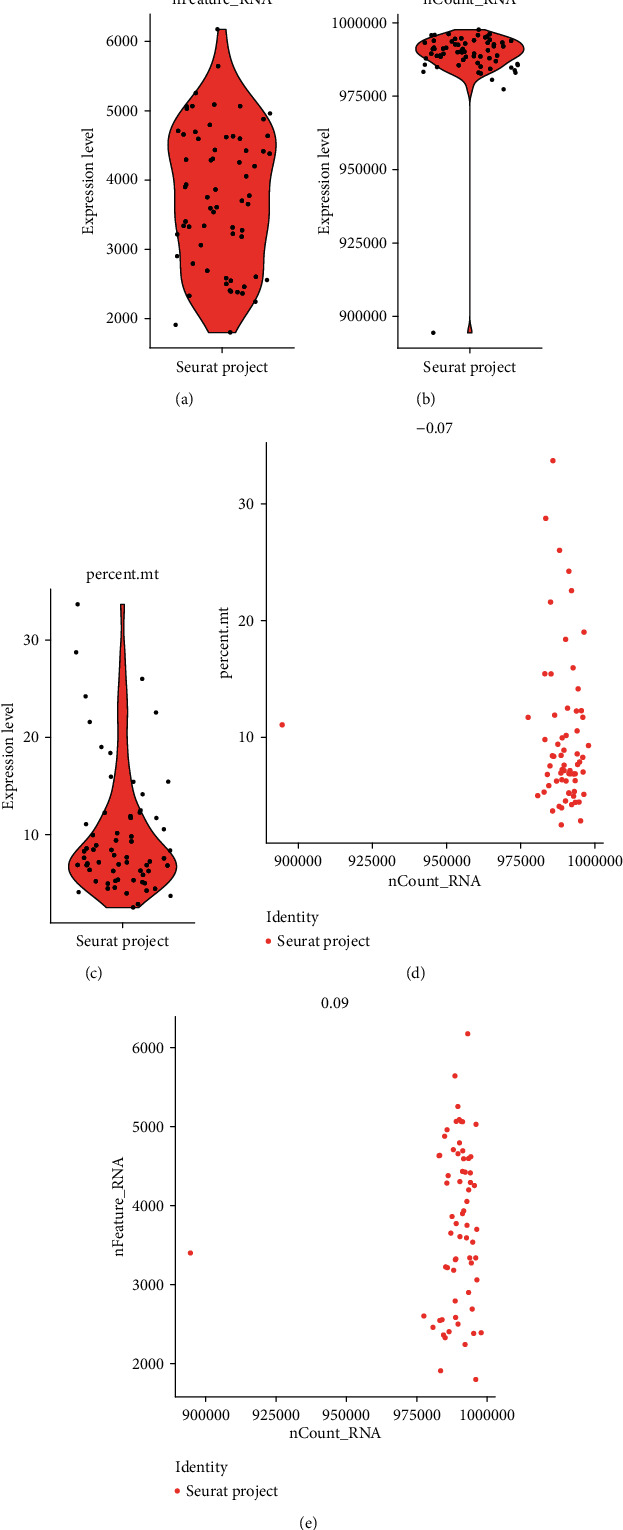
Quality control filtering to remove cells with low quality. (a) Violin plots showing the counts of genes in each cell. (b) Violin plots of the sum of the expression levels of all genes in each cell. (c) Violin plots of the percentage of mitochondrial genes. (d) Scatter plots for the percentage of mitochondrial genes in the sum of the expression levels of all genes in each cell. (e) Scatter plots for the counts of genes in the sum of the expression levels of all genes in each cell.

**Figure 2:**
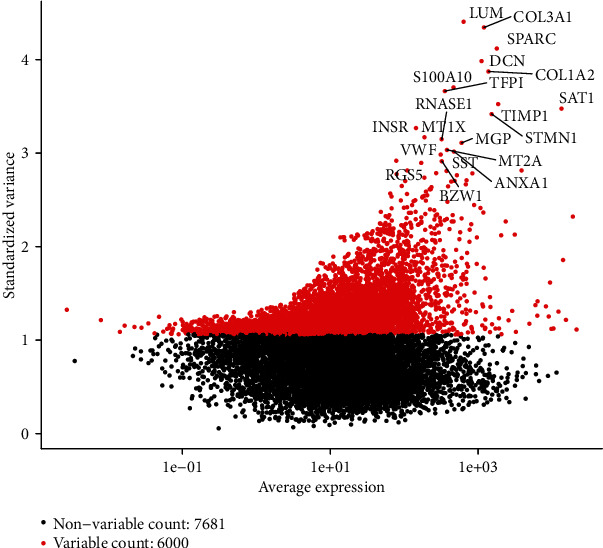
Detection of highly variable genes across the cells. *x* axis represents the average expression, and *y* axis represents standardized variance.

In total, 66 ovarian cancer cells were included in this study. Considering that the amount of data and the number of cells was relatively small, we used all the cells without filtering (Figures 1(a)–1(e)). Then, we detected 6000 highly variable genes across the single cells after calculating the mean and the variance to mean ratio of each gene. The top 20 highly variable genes such as LUM, COL3A1, and SPARC are shown in Figure 2.

**Figure 3:**
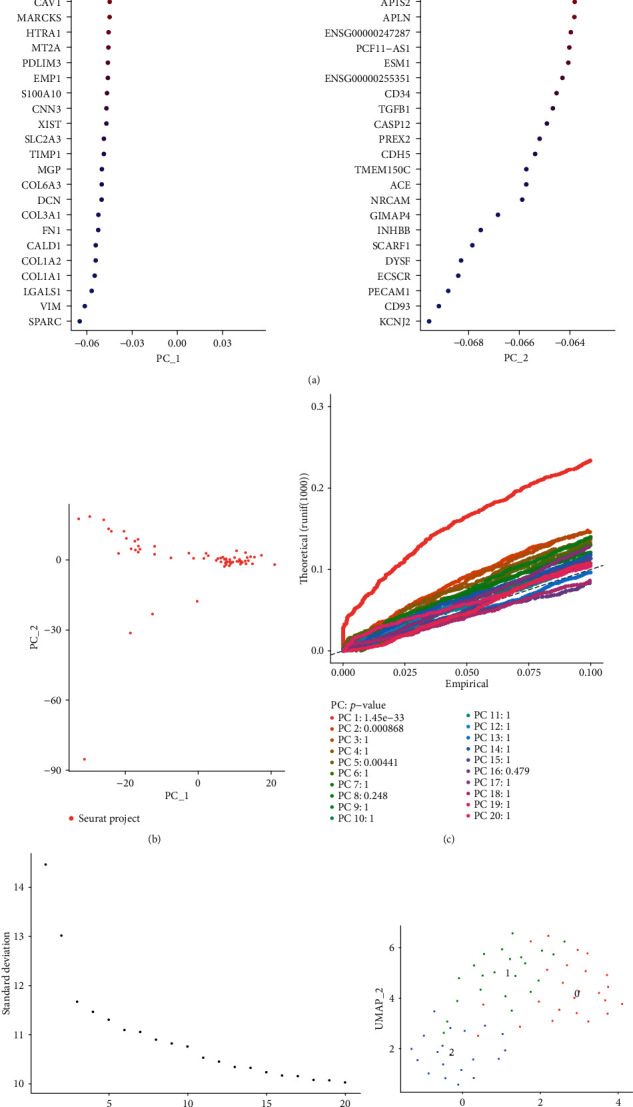
Individual principal component analysis. (a) The top 30 genes in PC1 and PC2. (b) The correlation between PC1 and PC2. (c) The *p* value of PCs. (d) The PCs were sorted based on the standard deviation. (e) UMAP plots showing the three cell clusters.

To overcome the various technical noise in any single feature of scRNA-seq data, the Seurat package was used to cluster cells according to their PCA scores, where each PC represented a “meta-feature” (Figures 3(a) and 3(b)). JackStraw function was used to resample the test. We randomly replaced a subset of the data (default was 1%) and rerun PCA to construct an “empty distribution” of feature scores and repeated the process (Figure 3(c)). We identified “important” PCs with low *p* values. Furthermore, the PCs were sorted based on the standard deviation using ElbowPlot function (Figure 3(d)). Because there was no obvious elbow point, we selected 19 PCs for downstream analysis. After cluster analysis, we divided the cells into 3 cell populations across ovarian cancer cells (Figure 3(e)). The number of cells in clusters 0, 1, and 2 was 24, 22, and 20. Supplementary table 2 listed which cells were in which cluster.

### 3.2. Analysis of Marker Genes in the Three Cell Subpopulations

**Figure 4:**
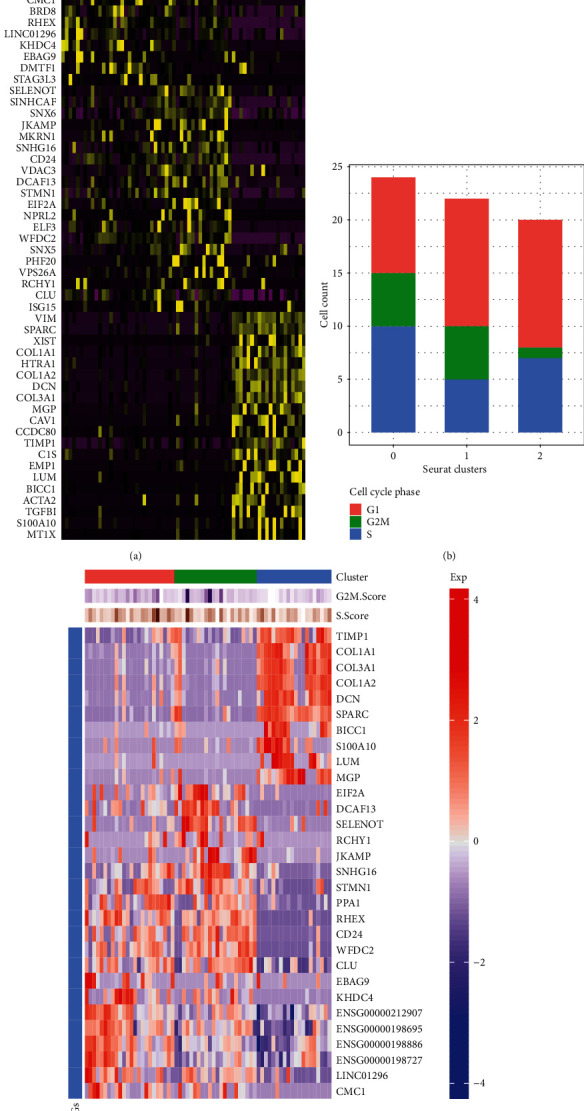
Cell cycle analyses. (a) The top 20 marker genes in the three cell subpopulations. (b) Cell cycle phase. (c) Cell cycle heatmap.

**Figure 5:**
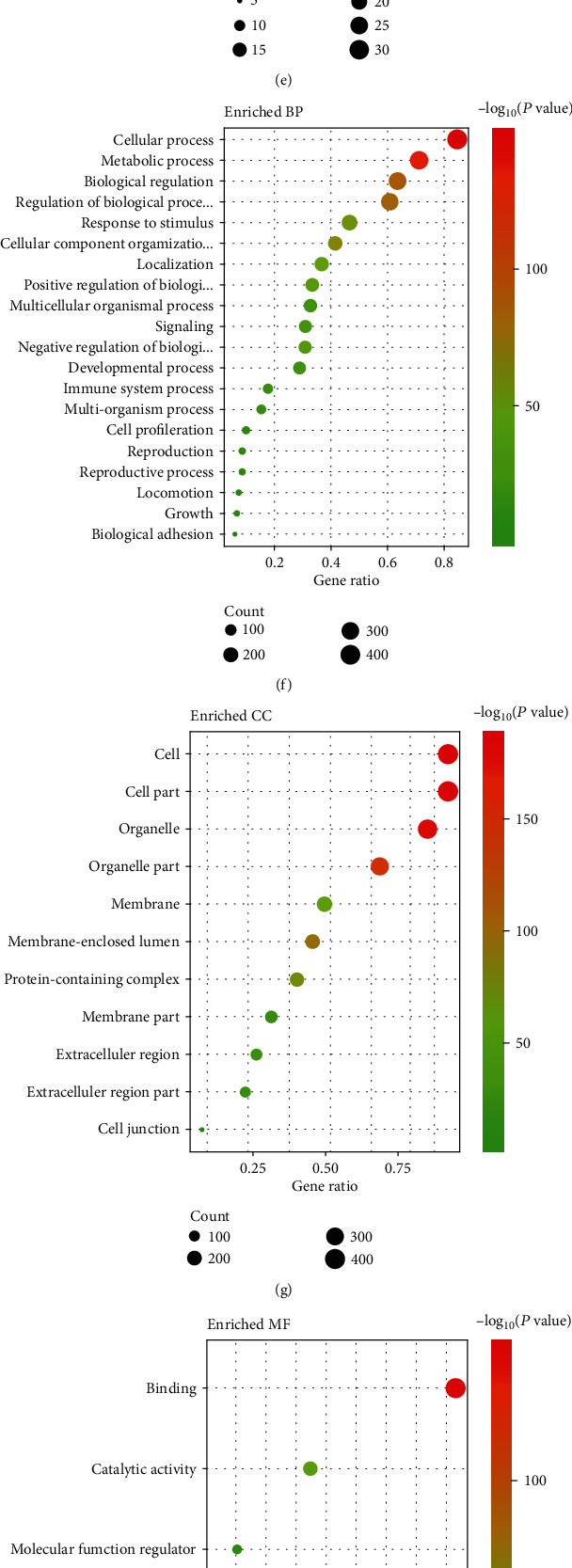
Function enrichment analyses. (a–d) The enriched KEGG pathways and GO terms including BP, CC, and MF in cluster 0. (e–h) The enriched KEGG pathways and GO terms including BP, CC, and MF in cluster 1. (i–l) The enriched KEGG pathways and GO terms including BP, CC, and MF in cluster 2.

The top 20 marker genes in the three cell subpopulations are listed in heatmap (Figure 4(a)). We used the Seurat tool to score the marker genes in the G1, G2M, and S cell cycles.  Figure 4(b) shows the cell counts in the G1, G2M, and S cell cycles. By Fisher's test, there was no significant difference between the three cell subpopulations and cells in each cell cycle (*p* value = 0.2834). Cell cycle heatmap shows the top ten differentially expressed genes and cell cycle scores in each cell subpopulation (Figure 4(c)). To explore potential biological processes and pathways, GO and KEGG enrichment analyses were performed (Figure 5). Genes in cluster 1 (Figures 5(a)–5(d)) and cluster 2 (Figures 5(e)–5(h)) were mainly enriched in metabolic processes and pathways. Meanwhile, genes in cluster 2 were primarily involved in cancer-related pathways such as PI3K-Akt pathway and pathways in cancer (Figures 5(i)–5(l)). We found that these marker genes were enriched in different biological processes and pathways in different cell subpopulations such as metabolic pathways, pathways in cancer, and mTOR signaling pathway.

### 3.3. Reconstruction of Differentiation Trajectories Using Monocle Package

**Figure 6:**
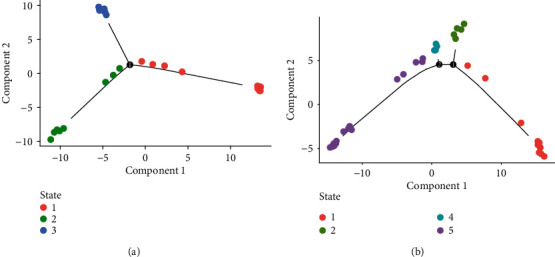
Reconstruction of differentiation trajectories to ovarian cancer. (a, b) The trajectory plot in pseudotime of epithelial cancer cells and stromal cells using Monocle analysis. Different colors represent different cell states.

Cell fate decisions and differentiation trajectories were reconstructed with the Monocle package. The pseudotime estimation analysis of epithelial cancer cells and stromal cells was performed based on the top 2000 highly variable marker genes (Figures 6(a) and 6(b)).

### 3.4. Identification of Differentially Expressed Genes Using TCGA Ovarian Cancer Datasets

**Figure 7:**
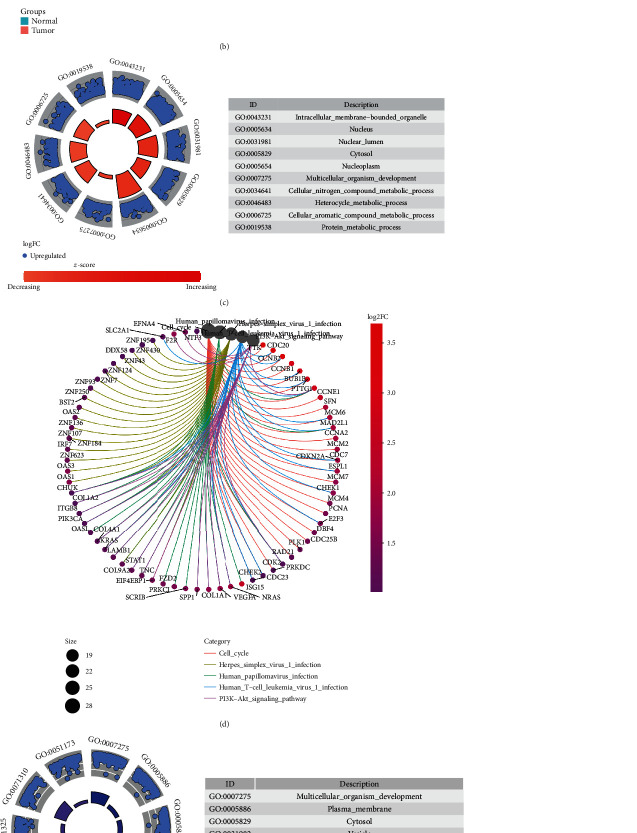
Differentially expressed genes of ovarian cancer. (a, b) Volcano plots and heatmap showing the differentially expressed genes with ∣log2FC | >1 and adjusted *p* value <0.05 between ovarian cancer and normal tissues, respectively. (c, d) GO and KEGG enrichment analysis results of upregulated genes. (e, f) GO and KEGG enrichment analysis results of downregulated genes.

A total of 1,124 differentially expressed genes with ∣log2FC | >1 and adjusted *p* value <0.05 were identified between 568 ovarian cancer samples and 8 normal samples (Figures 7(a) and 7(b)). GO enrichment analysis results showed that upregulated genes were primarily enriched in intracellular membrane-bounded organelle, nucleus, nuclear lumen, cytosol, nucleoplasm, cellular nitrogen compound metabolic process, heterocycle metabolic process, cellular aromatic compound metabolic process, and protein metabolic process (Figure 7(c)). Meanwhile, upregulated genes were involved in cell cycle, Herpes simplex virus 1 infection, human papillomavirus infection, human T cell leukemia virus 1 infection, and PI3K-Akt signaling pathway (Figure 7(d)). Downregulated genes primarily participated in multicellular organism development, plasma membrane, cytosol, vesicle, animal organ development, extracellular exosome, extracellular vesicle, positive regulation of cellular metabolic process, cellular response to organic substance, and positive regulation of nitrogen compound metabolic process (Figure 7(e)). In Figure 7(f), downregulated genes were mainly enriched in MAPK, metabolic, pathways in cancer, PI3K-Akt, and Ras signaling pathways.

### 3.5. Identification of Differentially Expressed Marker Genes Associated with Prognosis of Ovarian cancer

**Figure 8:**
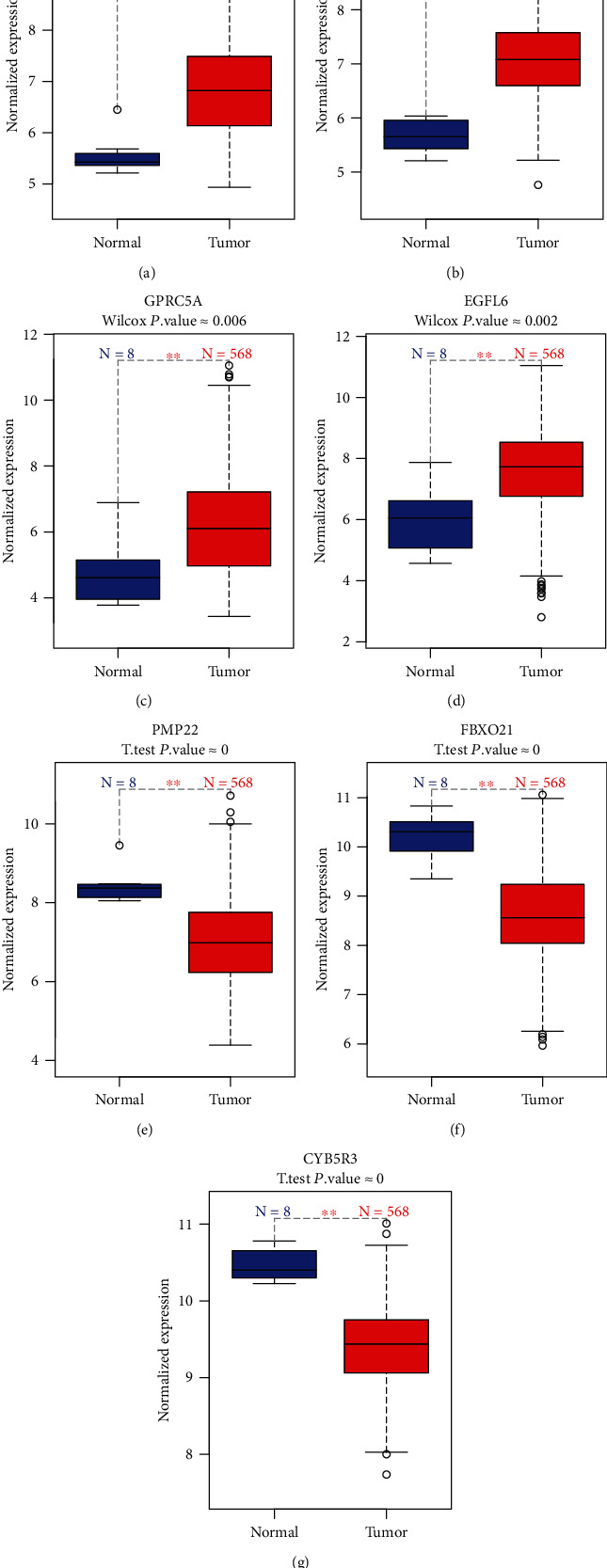
The differential expression of marker genes associated with prognosis of ovarian cancer. (a) STAT1; (b) ANP32E; (c) GPRC5A; (d) EGFL6; (e) PMP22; (f) FBXO21; (g) CYB5R3.

**Figure 9:**
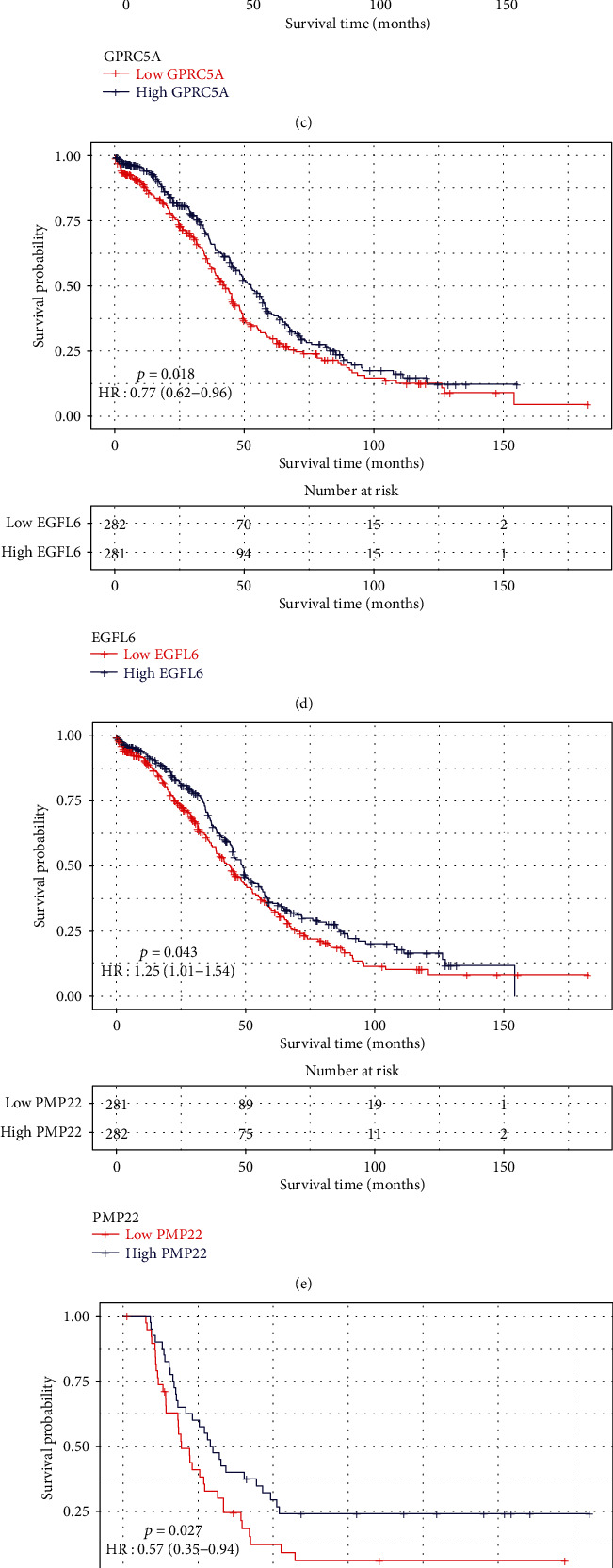
The survival analysis of differentially expressed marker genes in ovarian cancer. (a–e) The overall survival analysis results of ANP32E, STAT1, GPRC5A, EGFL6, and PMP22. (f–h) The recurrence-free survival analysis results of FBXO21, ANP32E, and CYB5R3.

**Figure 10:**
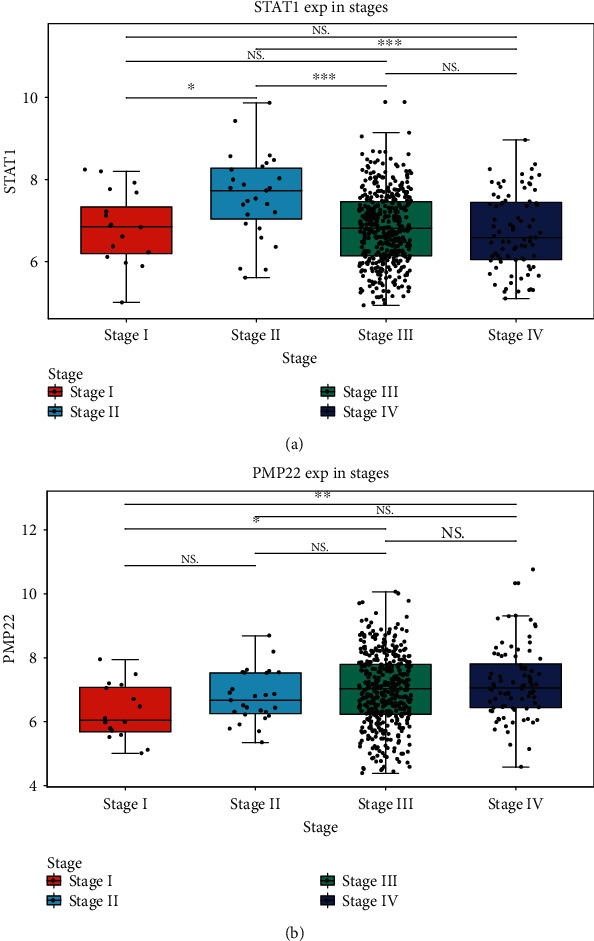
The differential expression of STAT1 and PMP22 across different stages in ovarian cancer. (a) STAT1; (b) PMP22.

All marker genes were overlapped with 1,124 differentially expressed genes in TCGA samples. Survival analysis was used for identifying prognosis-related differentially expressed marker genes. The results showed that marker genes STAT1, ANP32E, GPRC5A, and EGFL6 were highly expressed in ovarian cancer (Figures 8(a)–8(d)). Furthermore, marker genes PMP22, FBXO21, and CYB5R3 were lowly expressed in ovarian cancer (Figures 8(e)–8(g)). The high expression of ANP32E (*p* = 0.031, HR: 0.79 (0.64-0.98)), STAT1 (*p* = 0.005, HR: 0.74 (0.59-0.91)), GPRC5A (*p* = 0.03, HR: 1.27 (1.02-1.57)), EGFL6 (*p* = 0.018, HR: 0.77 (0.62-0.96)), and PMP22 (*p* = 0.043, HR: 1.25 (1.01-1.54)) was significantly associated with better overall survival time than their low expression (Figures 9(a)–9(e)). The high expression of FBXO21 (*p* = 0.027, HR: 0.57 (0.35-0.94)), ANP32E (*p* = 0.007, HR: 0.51 (0.31-0.84)), and CYB5R3 (*p* = 0.015, HR: 1.86 (1.12-3.08)) indicated better recurrence-free survival time compared with their low expression (Figures 9(f)–9(h)). Furthermore, we found that STAT1 had the highest expression in stage II among all stages (Figure 10(a)). PMP22 had the highest expression in stage III among all stages (Figure 10(b)).

## 4. Discussion

The treatment of ovarian cancer is complicated by the heterogeneity of the tumor. Different histological types of epithelial ovarian cancer have different cell origins, different mutation profiles, and different prognosis [16, 17]. Even in a histological type, different molecular subtypes with different prognosis can be found. To solve these problems, it is necessary to better characterize the heterogeneity of these ovarian cancer cells, to find reliable biomarkers, and develop appropriate targeted therapies. Single-cell RNA sequencing technology can explore the intercellular heterogeneity at the single-cell level and reconstruct lineage hierarchies. This method allows an unbiased analysis of the heterogeneity profile within a population of cells as it utilizes transcriptome reconstitution from a single cell. Our reanalysis of the ovarian cancer single-cell transcriptome may provide a deeper insight into the heterogeneity spectrum of ovarian cancer cells.

Totally, 66 ovarian cancer cells were included in our study. To remove cells with low quality, quality control was performed using the Seurat package. Proliferation induced by abnormal regulation of the cell cycle is thought to be critical for ovarian cancer progression. The G1/S phase is the most critical rate-limiting step in cell cycle promotion. Some studies have shown that the expression of cell cycle-related genes is significantly associated with poor prognosis in patients with ovarian cancer. Therefore, we studied molecules involved in cell cycle progression to discover new prognostic factors and therapeutic targets. In this study, 66 ovarian cancer cells were clustered into three groups (G1, G2M, and S). The marker genes in each cluster were identified. To explore potential biological processes and pathways, KEGG and GO enrichment analyses of these marker genes were performed. The results showed that the marker genes in each cluster were enriched in different biological processes and pathways.

Using ovarian cancer dataset from TCGA, a total of 1,124 differentially expressed genes with ∣log2FC | >1 and adjusted *p* value <0.05 were identified between 568 ovarian cancer tissues and 8 normal tissues. To explore potential biological processes and pathways, these differentially expressed genes were mainly enriched in metabolic pathways, pathways in cancer, PI3K-Akt signaling pathway, and the like. For example, most ovarian cancer cells are highly proliferative; therefore, they are highly dependent on the metabolism of glucose by the aerobic glycolysis or the Warburg effect [18, 19]. PI3K-Akt signaling pathway is deregulated in various malignant cancers including ovarian cancer, which participates in tumor cell proliferation, survival, metabolism, and angiogenesis [20, 21].

The intercellular heterogeneity is one of the major drivers of cancer progression [22]. Gene variation at the single-cell level can rapidly produce cancer heterogeneity [23]. Prognosis-related differentially expressed marker genes were identified. We found that the expression levels of STAT1, ANP32E, GPRC5A, and EGFL6 were all significantly higher in ovarian cancer tissues compared with normal tissues. Furthermore, PMP22, FBXO21, and CYB5R3 expression was significantly lower in ovarian cancer tissues compared with normal tissues. The low expression of ANP32E, STAT1, GPRC5A, EGFL6, and PMP22 was positively associated with overall survival time of ovarian cancer. The low expression of FBXO21, ANP32E, and CYB5R3 was significantly associated with longer recurrence-free survival time of ovarian cancer. STAT1, a member of STAT family, has been confirmed to be highly expressed in ovarian cancer [24, 25]. The high expression of ANP32E is in association with better prognosis, contributing to the proliferation and tumorigenesis of triple-negative breast cancer cells [26, 27]. GPRC5A variants may drive self-renewal of bladder cancer stem cells according to single-cell RNA-seq analysis [28]. EGFL6, a stem cell regulator expressed in ovarian tumor cells and vasculature, may induce the growth and metastasis of ovarian cancer [29, 30]. A previous study has found that EGFL6 is upregulated in drug-resistant ovarian cancer cell lines using microarray analysis [31]. The expression and function of PMP22 in tumors remain unclear. Some studies have shown that PMP22 is a potential tumor suppressor, and others have indicated that PMP22 has a potential carcinogenic function in tumors [32–35]. Studies on the role of PMP22 in the regulation of ovarian cancer have not been reported. Furthermore, there is no report concerning the expression and role of FBXO21 and CYB5R3 in ovarian cancer. Collectively, our study identified specific cell subpopulations and marker genes in ovarian cancer.

## 5. Conclusion

In our study, we analyzed the intercellular heterogeneity in ovarian cancer using single-cell RNA sequencing and identified marker genes in each cluster. Combining TCGA ovarian cancer dataset, we identified differentially expressed marker genes that were significantly associated with prognosis of ovarian cancer, including ANP32E, STAT1, GPRC5A, EGFL6, PMP22, FBXO21, and CYB5R3.

## Data Availability

The data used to support the findings of this study are included within the supplementary information files.

## Abbreviations

RNA-seq: RNA sequencing
GEO: Gene Expression Omnibus
PCA: Principal component analysis
TCGA: The Cancer Genome Atlas
FC: Fold change
UMAP: Uniform Manifold Approximation and Projection
GO: Gene Ontology
KEGG: Kyoto Encyclopedia of Genes and Genomes
BP: Biological process
CC: Cellular component
MF: Molecular function.

## Supplementary Materials

Supplementary 1 Supplementary table 1: the IDs of TCGA ovarian cancer samples.

Supplementary 2 Supplementary table 2: the list of genes in each cluster.
