# Supplementary material for: Identification of Specific Cell Subpopulations and Marker Genes in Ovarian Cancer Using Single-Cell RNA Sequencing
Source: Biomed Res Int. 2021 Oct 7;2021:1005793. doi: 10.1155/2021/1005793 (PMC8517627; doi:10.1155/2021/1005793)
Supplement: Supplementary 1 — Supplementary table 1: the IDs of TCGA ovarian cancer samples. [file 1005793.f1.pdf]

Supplementary table 1. The IDs of TCGA ovarian cancer samples.

Group	Sample
0	X1.C01
0	X1.C15
0	X1.C19
0	X1.C22
0	X1.C31
0	X1.C34
0	X1.C37
0	X1.C38
0	X1.C40
0	X1.C81
0	X2.C05
0	X2.C14
0	X2.C16
0	X2.C21
0	X2.C25
0	X2.C29
0	X2.C34
0	X2.C49
0	X2.C57
0	X2.C69
0	X2.C73
0	X2.C79
0	X2.C87
0	X2.C95
1	X1.C03
1	X1.C08
1	X1.C17
1	X1.C18
1	X1.C23
1	X1.C27
1	X1.C29
1	X1.C51
1	X1.C61
1	X1.C75
1	X2.C06
1	X2.C18
1	X2.C19
1	X2.C23
1	X2.C42
1	X2.C51
1	X2.C55
1	X2.C70
1	X2.C72
1	X2.C74
1	X2.C75
1	X2.C86
2	X1.C02
2	X1.C30
2	X1.C41
2	X1.C42
2	X1.C49

2	X1.C65
2	X1.C67
2	X2.C03
2	X2.C07
2	X2.C10
2	X2.C15
2	X2.C22
2	X2.C27
2	X2.C53
2	X2.C56
2	X2.C59
2	X2.C63
2	X2.C64
2	X2.C67
2	X2.C71
