# Supplementary material for: Identification of Specific Cell Subpopulations and Marker Genes in Ovarian Cancer Using Single-Cell RNA Sequencing
Source: Biomed Res Int. 2021 Oct 7;2021:1005793. doi: 10.1155/2021/1005793 (PMC8517627; doi:10.1155/2021/1005793)
Supplement: Supplementary 2 — Supplementary table 2: the list of genes in each cluster. [file 1005793.f2.pdf]

Supplementary table 2. The list of which cells were in which cluster.
TCGA-61-1910-01
TCGA-61-1728-01
TCGA-13-1819-02
TCGA-31-1953-01
TCGA-13-1819-01
TCGA-13-0764-01
TCGA-31-1951-01
TCGA-24-1552-01
TCGA-13-1477-01
TCGA-30-1862-01
TCGA-29-1698-01
TCGA-13-0924-01
TCGA-25-1326-01
TCGA-09-1659-01
TCGA-23-1021-01
TCGA-04-1525-01
TCGA-23-2084-01
TCGA-24-2281-01
TCGA-61-1918-01
TCGA-24-0982-01
TCGA-04-1516-01
TCGA-23-1122-01
TCGA-23-1109-01
TCGA-01-0628-11
TCGA-24-1924-01
TCGA-09-1666-01
TCGA-61-1724-01
TCGA-24-1426-01
TCGA-23-1116-01
TCGA-25-2398-01
TCGA-25-2409-01
TCGA-29-1778-01
TCGA-13-0791-02
TCGA-04-1343-01
TCGA-13-0791-01
TCGA-13-1506-01
TCGA-04-1360-01
TCGA-01-0639-11
TCGA-42-2582-01
TCGA-13-1496-01
TCGA-25-1626-01
TCGA-20-0987-01
TCGA-09-1662-01
TCGA-24-2024-01
TCGA-01-0630-11
TCGA-09-1675-01
TCGA-04-1644-01
TCGA-10-0931-01
TCGA-25-1623-01
TCGA-29-1774-01
TCGA-61-2016-01
TCGA-13-0795-01

TCGA-61-2113-01
TCGA-13-1492-01
TCGA-24-2260-01
TCGA-13-1485-01
TCGA-13-2057-01
TCGA-36-2552-01
TCGA-23-1024-01
TCGA-09-0365-01
TCGA-13-0885-01
TCGA-29-1688-01
TCGA-24-1928-01
TCGA-25-1315-01
TCGA-13-0804-01
TCGA-25-1877-01
TCGA-36-2542-01
TCGA-24-2297-01
TCGA-61-2111-01
TCGA-23-1119-01
TCGA-20-1687-01
TCGA-13-1489-02
TCGA-13-1489-01
TCGA-29-1694-01
TCGA-30-1892-01
TCGA-25-1319-01
TCGA-04-1349-01
TCGA-24-1564-01
TCGA-61-1722-01
TCGA-20-1683-01
TCGA-24-2038-01
TCGA-23-1031-01
TCGA-13-0755-01
TCGA-04-1652-01
TCGA-36-1575-01
TCGA-36-1568-01
TCGA-31-1959-01
TCGA-57-1993-01
TCGA-23-2081-01
TCGA-09-0369-01
TCGA-09-2053-01
TCGA-24-1556-01
TCGA-25-1323-01
TCGA-13-1512-01
TCGA-09-0364-01
TCGA-13-0802-01
TCGA-29-1761-01
TCGA-61-1738-01
TCGA-25-2400-01
TCGA-29-2434-01
TCGA-13-0751-01
TCGA-29-2436-01
TCGA-61-1899-01
TCGA-13-1404-01
TCGA-57-1585-01

TCGA-59-2351-01
TCGA-04-1335-01
TCGA-13-0894-01
TCGA-04-1356-01
TCGA-61-1734-01
TCGA-24-1423-01
TCGA-13-0766-01
TCGA-25-1632-01
TCGA-29-1770-01
TCGA-13-1408-01
TCGA-29-1770-02
TCGA-61-1730-01
TCGA-29-1691-01
TCGA-61-2101-01
TCGA-24-1845-01
TCGA-04-1365-01
TCGA-59-2355-01
TCGA-25-1634-01
TCGA-30-1860-01
TCGA-10-0927-01
TCGA-13-0890-01
TCGA-24-1544-01
TCGA-24-1849-01
TCGA-13-1410-01
TCGA-36-1571-01
TCGA-13-0920-01
TCGA-13-0714-01
TCGA-24-2293-01
TCGA-13-2071-01
TCGA-24-0968-01
TCGA-04-1530-01
TCGA-24-1553-01
TCGA-13-2065-01
TCGA-24-1603-01
TCGA-36-1581-01
TCGA-61-2613-01
TCGA-13-0727-01
TCGA-23-1123-01
TCGA-04-1369-01
TCGA-61-2000-01
TCGA-23-1111-01
TCGA-04-1337-01
TCGA-30-1859-01
TCGA-61-2002-01
TCGA-25-2404-01
TCGA-29-1702-01
TCGA-29-1710-02
TCGA-24-1466-01
TCGA-36-2539-01
TCGA-04-1348-01
TCGA-13-0919-01
TCGA-61-2009-01
TCGA-10-0933-01

TCGA-61-1995-01
TCGA-24-1427-01
TCGA-24-1847-01
TCGA-09-1670-01
TCGA-30-1855-01
TCGA-04-1648-01
TCGA-09-2056-01
TCGA-04-1361-01
TCGA-13-0794-01
TCGA-24-1551-01
TCGA-13-0765-01
TCGA-09-2044-01
TCGA-24-2035-01
TCGA-09-1674-01
TCGA-13-0884-01
TCGA-13-1497-01
TCGA-01-0631-11
TCGA-20-1685-01
TCGA-10-0936-01
TCGA-13-0913-02
TCGA-13-0913-01
TCGA-24-1923-01
TCGA-13-1484-01
TCGA-23-1114-01
TCGA-24-1471-01
TCGA-61-2097-01
TCGA-13-2059-01
TCGA-42-2588-01
TCGA-61-2088-01
TCGA-04-1367-01
TCGA-25-2042-01
TCGA-24-1920-01
TCGA-61-1916-02
TCGA-61-1916-01
TCGA-61-2096-01
TCGA-09-1667-01
TCGA-36-1569-01
TCGA-24-2029-01
TCGA-36-2548-01
TCGA-29-2431-01
TCGA-09-1669-01
TCGA-29-1776-01
TCGA-04-1341-01
TCGA-25-1871-01
TCGA-13-0923-01
TCGA-24-2020-01
TCGA-04-1353-01
TCGA-04-1655-01
TCGA-25-1313-01
TCGA-23-2643-01
TCGA-13-0720-01
TCGA-04-1357-01
TCGA-13-0899-01

TCGA-13-0768-01
TCGA-59-2350-01
TCGA-29-2428-01
TCGA-24-1557-01
TCGA-24-1435-01
TCGA-36-2540-01
TCGA-13-0889-01
TCGA-59-2354-01
TCGA-13-0803-01
TCGA-01-0642-11
TCGA-24-1413-01
TCGA-61-2109-01
TCGA-04-1514-01
TCGA-29-1690-01
TCGA-04-1338-01
TCGA-24-1555-01
TCGA-61-2098-01
TCGA-29-1769-01
TCGA-36-2544-01
TCGA-25-1325-01
TCGA-24-1567-01
TCGA-24-2023-01
TCGA-57-1583-01
TCGA-24-1474-01
TCGA-36-2530-01
TCGA-23-1032-01
TCGA-57-1584-01
TCGA-42-2590-01
TCGA-13-0906-01
TCGA-61-1903-01
TCGA-59-2352-01
TCGA-24-1563-01
TCGA-61-2612-01
TCGA-24-0979-01
TCGA-13-1500-01
TCGA-36-2534-01
TCGA-25-1625-01
TCGA-24-1467-01
TCGA-04-1332-01
TCGA-13-0792-01
TCGA-13-1510-01
TCGA-24-0970-01
TCGA-25-1320-01
TCGA-13-1501-01
TCGA-09-0367-01
TCGA-24-0975-01
TCGA-13-0908-01
TCGA-59-2349-01
TCGA-25-1317-01
TCGA-25-1635-01
TCGA-13-0762-01
TCGA-23-1028-01
TCGA-25-2408-01

TCGA-25-1329-01
TCGA-61-2008-01
TCGA-61-2008-02
TCGA-24-1546-01
TCGA-25-1630-01
TCGA-04-1649-01
TCGA-61-1913-01
TCGA-20-0991-01
TCGA-13-1505-01
TCGA-09-2048-01
TCGA-36-1574-01
TCGA-30-1861-01
TCGA-30-1856-01
TCGA-24-1417-01
TCGA-61-2610-02
TCGA-24-1614-01
TCGA-13-1495-01
TCGA-59-2372-01
TCGA-13-0904-01
TCGA-23-1121-01
TCGA-36-2529-01
TCGA-23-1023-01
TCGA-13-2061-01
TCGA-36-2551-01
TCGA-30-1891-01
TCGA-29-2414-01
TCGA-29-2414-02
TCGA-13-0912-01
TCGA-24-1843-01
TCGA-24-1470-01
TCGA-36-1580-01
TCGA-13-0916-01
TCGA-42-2589-01
TCGA-20-1684-01
TCGA-13-1488-01
TCGA-09-2050-01
TCGA-30-1880-01
TCGA-30-1867-01
TCGA-61-1901-01
TCGA-29-1710-01
TCGA-13-0901-01
TCGA-61-2018-01
TCGA-57-1992-01
TCGA-04-1351-01
TCGA-61-1736-01
TCGA-10-0937-01
TCGA-09-1665-01
TCGA-36-2538-01
TCGA-24-1852-01
TCGA-23-2641-01
TCGA-61-1740-01
TCGA-61-1900-01
TCGA-13-1499-01

TCGA-29-1781-01
TCGA-61-1915-01
TCGA-29-1704-01
TCGA-24-1616-01
TCGA-61-1727-01
TCGA-29-2427-01
TCGA-25-1870-01
TCGA-29-2425-01
TCGA-09-1672-01
TCGA-24-1550-01
TCGA-13-0886-01
TCGA-29-1763-01
TCGA-24-2288-01
TCGA-25-1312-01
TCGA-09-2055-01
TCGA-10-0926-01
TCGA-13-0807-01
TCGA-13-1482-01
TCGA-31-1956-01
TCGA-13-0730-01
TCGA-04-1519-01
TCGA-61-1737-01
TCGA-23-2645-01
TCGA-61-2104-01
TCGA-13-0801-01
TCGA-23-2647-01
TCGA-13-0888-01
TCGA-24-2033-01
TCGA-10-0934-01
TCGA-29-1764-01
TCGA-29-1696-01
TCGA-04-1654-01
TCGA-04-1536-01
TCGA-61-2095-02
TCGA-23-1027-01
TCGA-04-1651-01
TCGA-61-1906-01
TCGA-04-1346-01
TCGA-57-1994-01
TCGA-25-2399-01
TCGA-24-1548-01
TCGA-36-2545-01
TCGA-36-1577-01
TCGA-24-1562-01
TCGA-24-1463-01
TCGA-61-1725-01
TCGA-13-1403-01
TCGA-24-1431-01
TCGA-36-1578-01
TCGA-24-2262-01
TCGA-04-1342-01
TCGA-04-1336-01
TCGA-36-2549-01

TCGA-29-1784-01
TCGA-42-2591-01
TCGA-29-1777-01
TCGA-09-1661-01
TCGA-29-2429-01
TCGA-23-2072-01
TCGA-24-1565-01
TCGA-24-0980-01
TCGA-13-0903-01
TCGA-13-1511-01
TCGA-24-1425-01
TCGA-25-2392-01
TCGA-29-1701-01
TCGA-24-1846-01
TCGA-24-2027-01
TCGA-09-0366-01
TCGA-24-1850-01
TCGA-09-2045-01
TCGA-24-1852-02
TCGA-25-1316-01
TCGA-61-1733-01
TCGA-20-0990-01
TCGA-59-2348-01
TCGA-23-1118-01
TCGA-13-0726-01
TCGA-13-0793-01
TCGA-61-2095-01
TCGA-13-0897-01
TCGA-24-1103-01
TCGA-13-1817-02
TCGA-13-1498-01
TCGA-24-2298-01
TCGA-25-1328-01
TCGA-25-1631-01
TCGA-13-0725-01
TCGA-24-2019-01
TCGA-13-0893-01
TCGA-57-1582-01
TCGA-09-2049-01
TCGA-13-0910-01
TCGA-31-1946-01
TCGA-23-1124-01
TCGA-25-1321-01
TCGA-29-1692-02
TCGA-29-1768-01
TCGA-29-1692-01
TCGA-30-1857-01
TCGA-13-0900-01
TCGA-24-1416-01
TCGA-25-1324-01
TCGA-04-1364-01
TCGA-23-1107-01
TCGA-01-0636-11

TCGA-61-2092-01
TCGA-24-1422-01
TCGA-23-1022-01
TCGA-24-1428-01
TCGA-36-2532-01
TCGA-13-2060-01
TCGA-61-2614-01
TCGA-24-2254-01
TCGA-23-1120-01
TCGA-23-1113-01
TCGA-42-2587-01
TCGA-13-1504-01
TCGA-24-2290-01
TCGA-09-1668-01
TCGA-29-1783-01
TCGA-13-1407-01
TCGA-24-1424-01
TCGA-13-1507-01
TCGA-24-1105-01
TCGA-13-0724-01
TCGA-24-1469-01
TCGA-61-1917-01
TCGA-36-1570-01
TCGA-25-1322-01
TCGA-23-2079-01
TCGA-09-1664-01
TCGA-01-0637-11
TCGA-13-0758-01
TCGA-13-0883-01
TCGA-13-1412-01
TCGA-61-1741-01
TCGA-04-1646-01
TCGA-24-1434-01
TCGA-25-1633-01
TCGA-25-2396-01
TCGA-61-1914-01
TCGA-29-1705-01
TCGA-24-2036-01
TCGA-29-1705-02
TCGA-13-0797-01
TCGA-13-2066-01
TCGA-04-1362-01
TCGA-13-1509-01
TCGA-13-1494-01
TCGA-04-1350-01
TCGA-09-1673-01
TCGA-25-1627-01
TCGA-29-1785-01
TCGA-61-2087-01
TCGA-61-1911-01
TCGA-61-2012-01
TCGA-13-0717-01
TCGA-29-1699-01

TCGA-24-1930-01
TCGA-13-0887-01
TCGA-31-1950-01
TCGA-57-1586-01
TCGA-24-2271-01
TCGA-13-1491-01
TCGA-24-2267-01
TCGA-04-1517-01
TCGA-61-1904-01
TCGA-13-1483-01
TCGA-10-0938-01
TCGA-30-1718-01
TCGA-13-0921-01
TCGA-24-2280-01
TCGA-13-0799-01
TCGA-13-1487-01
TCGA-24-1604-01
TCGA-29-1695-01
TCGA-29-1697-01
TCGA-25-1318-01
TCGA-36-1576-01
TCGA-13-1411-01
TCGA-30-1887-01
TCGA-30-1714-01
TCGA-23-1026-01
TCGA-13-0757-01
TCGA-09-2054-01
TCGA-61-1919-01
TCGA-61-2017-01
TCGA-24-1558-01
TCGA-23-1117-01
TCGA-09-2051-01
TCGA-29-1711-01
TCGA-23-1809-01
TCGA-24-1418-01
TCGA-23-2077-01
TCGA-24-1430-01
TCGA-61-1895-01
TCGA-04-1347-01
TCGA-20-0996-01
TCGA-10-0930-01
TCGA-13-1817-01
TCGA-29-1775-01
TCGA-24-0966-01
TCGA-61-2102-01
TCGA-24-2261-01
TCGA-61-2003-01
TCGA-13-0800-01
TCGA-25-2391-01
TCGA-25-1314-01
TCGA-25-2393-01
TCGA-13-1409-01
TCGA-29-1762-01

TCGA-24-2026-01
TCGA-10-0928-01
TCGA-13-0805-01
TCGA-61-1998-01
TCGA-23-1029-01
TCGA-10-0925-01
TCGA-61-1721-01
TCGA-36-2543-01
TCGA-61-2110-01
TCGA-20-1686-01
TCGA-24-1549-01
TCGA-13-0761-01
TCGA-29-1766-01
TCGA-59-2363-01
TCGA-25-1878-01
TCGA-24-1419-01
TCGA-24-1560-01
TCGA-23-1030-01
TCGA-25-2401-01
TCGA-29-1693-01
TCGA-61-2611-02
TCGA-20-1682-01
TCGA-24-2295-01
TCGA-30-1866-01
TCGA-10-0935-01
TCGA-24-2030-01
TCGA-29-2432-01
TCGA-13-0905-01
TCGA-04-1331-01
TCGA-30-1853-01
TCGA-13-0911-01
TCGA-36-2533-01
TCGA-23-1110-01
TCGA-24-1842-01
TCGA-13-1405-01
TCGA-09-2043-01
TCGA-24-1464-01
TCGA-29-1703-01
TCGA-36-2537-01
TCGA-61-1743-01
TCGA-42-2593-01
TCGA-24-1104-01
TCGA-23-2649-01
TCGA-31-1944-01
TCGA-29-1771-01
TCGA-04-1542-01
TCGA-61-2094-01
TCGA-25-1628-01
TCGA-04-1371-01
TCGA-29-1707-02
TCGA-29-1707-01
TCGA-24-1844-01
TCGA-24-1436-01

TCGA-23-2078-01
TCGA-13-0891-01
TCGA-24-2289-01
TCGA-13-1481-01
TCGA-25-2397-01
TCGA-24-1545-01
TCGA-61-1907-01
TCGA-01-0633-11
TCGA-04-1638-01
TCGA-36-2547-01
TCGA-13-0723-01
